# Abstracts and Gleanings

**Published:** 1879-02-20

**Authors:** 


### ABSTRACTS AND GLEANINGS.

**Alkaloids of Opium—Their Action.**—The well-determined opium alkaloids now number sixteen. The effect of any one differs from the rest or from that of opium itself. Dr. Isaac Ott (*Journal Nervous and Mental Diseases*, January, 1878), reports a large number of experiments which, added to our previous knowledge, enable him to draw the following conclusions :

1. *Cryptopia* is narcotic; excites and then depresses reflex action by an effect on the spinal cord, reduces power of motor nerves, abolishes sensation by an action on the spinal sensory ganglia, and lowers the heart-beat by an action on its muscular structure.

2. *Thebaine* is a spinal convulsivant; has no action on motor or sensory nerves or striated muscle. It reduces the heart-beat by an action on that organ, and increased blood pressure by stimulating the cerebral vaso-motor centers.

3. *Codeia* is a narcotic and spinal convulsivant; produces a veratroid contraction of striated muscles and depresses the heart-beat by an action on the cardiac muscle.

4. *Chlorocodide* is a tetanic agent.

5. *Apocodeia* produces vomiting, coma and death.

6. *Narceine* is soporific to cold-blooded animals but not to man, and is a spinal convulsivant. It does not destroy the motor nerves, as they act on thrusting a probe down the spine. It produces veratroid contraction of the muscle, and reduces the heart-beat by stimulation of the peripheral end of the pneumogastric.

7. *Papaverine* is narcotic and convulsivant; the convulsions being partly spinal and partly peripheral, the latter, it is highly probable, from an action on the muscle. It diminishes the heart's contractions by peripheral action on the cardio-inhibitory apparatus. It also causes veratroid contraction of the muscle.

8. *Narcotine* is non-narcotic, and a spinal convulsivant; produces veratroid contraction of striated muscle and is a very active agent to decrease the beats of the heart by an action on cardiac muscle.

9. *Cotarnine* is soporific, and paralyses, like curare, the motor nerve.

10. *Hydro-cotarnine* is narcotic and convulsivant.

11. *Hydro-chlorate of Cotarnamic Acid* is a convulsivant, and paralyzes the pneumogastric.

12. *Laudanosine* and *Laudanine* are tetanic agents.

13. *Morphia* is a narcotic and spinal convulsivant, It produces veratroid contraction of muscle and reduces the heart-beat.

14. *Oxymorphia* has an action like morphia, only weaker.

15. *Apomorphia* is an emetic; excites and reduces spinal reflex excitability, and diminishes the number of cardiac contractions.

16. *Meconine* is narcotic to cold-blooded animals, but not in doses of two grains by the stomach in man. It causes hyperæsthesia and paralysis of voluntary motion with general relaxation. It also produces a veratroid contraction.

The opium alkaloids all have a dominant action on the nervous system, causing first increased, exaggerated functions and, if the dose is large enough, a paralysis of them. In the warm-blooded animals this action is both on the spinal cord and cerebrum.—*Detroit Lancet.*

**Oxalate of Cerium in Chronic Cough.**—Dr. James G. La Roe, Jr., of Greenpoint, L. I., writes: "In the *Medical Record* bearing date May 25, 1878, and credited to the *Practitioner*, is a short article on the use of oxalate of cerium in chronic cough. It is highly lauded by Thomas Clark, L.R.C.P., etc. In doses of five grains, we are told, it is a good sedative, and hence its usefulness in lung troubles. Its virtues in stomach complaints are fully appreciated. In the intractable vomiting of pregnancy I have found it the remedy *par excellence.* It struck me, however, that the dose was rather large. Two-grain doses, in my hands, have done the tranquilizing work desired. I had been treating a lady whose phthisical cough was a source of great worriment to me. Everything suitable to the case was tried, but to no effect. The cough was incessant, and wore her completely down. The drugs which had served her so well hitherto refused now to give her even partial rest. As I never tried it in lung troubles, I determined to give the oxalate of cerium strictly according to directions. Five grains were ordered to be taken a half hour before rising. The result was, my patient was unable to get out of bed until late in the afternoon. That the drug was a sedative admitted of no doubt, for Mrs. L. did not gain her full senses until the time indicated. She described herself as having been in a stupor all day. The reaction, seemingly, made her cough as bad, if not worse than before. Altogether, the effect was rather unpleasant. Of course, her memory as to whether she coughed meanwhile went for nothing. It was beyond her power to grasp any fact until the profound sedative action had passed away. I was anxious to see if these feelings would be repeated, and so prevailed on her to try once more. The same result occurred again. That the dose was too large was very manifest. Equally suggestive was the thought that its true vocation was in a smaller quantity. It required not a little persuasion to get Mrs. L. to try still further. I only succeeded after declaring I would desist after this trial. I directed half a powder to be taken in the same manner as before. The sequela was all that could be desired; I was fully repaid for my persistency. My patient did not cough the remainder of the day, and felt very grateful in consequence. She took the medicine several times more, not waiting to be told to do so. Since then, the oxalate of cerium has done duty several times in Mrs. L.'s case, and with the greatest satisfaction, as before.

"In one other case, within a few weeks past, and of precisely the same nature, I ordered the drug to be taken in three-grain doses. The *finale* was in every way gratifying."—*N. Y. Med. Rec.*

**Brunella Vulgaris.**—This plant is known by the common names of self-heal, heal-all, or wild hop, belonging to the mint family, growing by the wayside in low ground, and quite plentifully in the northern part of Ohio. It flowers in July and August with a blue blow. The plant is the part used. It is not used as a medicinal agent by the profession in the United States, as far as I can ascertain. I have used it extensively for three years, and find it to contain medical properties of great value.

My attention was first called to the plant in a case of profuse flow of lochia after labor, that gave me a great deal of trouble, and would not yield to the old treatment, but immediately stopped when put on an infusion of the brunella. I have since used it in many cases of excessive menstruation, where the discharge is of a light color. I use it in uterine leucorrhœa internally, and all diseases peculiar to the female, and think I know what I am saying when I say it has no equal in discharges of mucus mixed with blood.

In an obstinate case of gonorrhæa in a male that fell into my hands to treat, the brunella cured in a short time by giving internally. And now I speak of the disease that so many are glad to get relief from, if not a permanent cure, catarrh. I am treating cases of this disease with the plant to my entire satisfaction, by using internally and as a wash. The dose that I use is :

Brunella.................. ʒ j.
Water..................... ℥ iv.

A teaspoonful every three hours. Of an infuson :

Brunella.................. ʒ ss.

Boiling water............. Oj.

Dose f℥ss. every three hours.

I have used it mostly in infusion, but have good results from the tincture.

I have given a few hurried cases in which I have used this plant, not from any selfish motive of my own, but for the good of the profession, hoping it may be the means of relieving some poor sufferer.—*Dr. Reynolds.*—*Ec. Med. Journal.*

**Tubercular Meningitis in the Adult.**—The more ordinary symptoms that mark the invasion of tubercular meningitis in the adult, may be summarized as follows :

1. *Headache* is the most invariable symptom, seldom, if ever, absent.

2. *Vomiting, constipation* and *fever* are present, attended by no characteristic rash.

3. Peculiarity of temper and conduct, occasional confusion of ideas and slight transitory delirium, are also symptomatic of this disease.

4. General muscular pains, followed first by stiffness and then by slight paralysis, as shown in the imperfect co-ordination of the muscular movements in tremblings and in twitchings. The pain and stiffness are first felt in the nape of the neck, and then in the muscles of the back.

5. *Slight epileptiform convulsions are observed, followed by paralysis of the limbs or parts convulsed;* this paralysis being most usually of a transitory or temporary kind. Among the characteristic and most frequently noticed paralyses are those affecting the optic commissure and oculomotor tracts, causing a slight internal squint, with dilated inactive pupil of one eye, with drooping of the same eyelid and paralysis of the facial nerve upon one side. The paralysis of the limbs is usually a hemiplegia: still it seldom affects all the muscles and its progress is gradual. Co-ordination of movement is seldom perfect in the limbs which recover.

6. Hyperæsthesia of the skin generally appears, with peculiar mental phenomena; as obstinate and unaccommodating conduct, altered temper and attitude of dogged resistence. Very little nourishment is voluntarily taken. The retracted abdomen and the aspect of the patient, with half-open eyelids or some slight paralysis of these becomes highly diagnostic.

7. *Continual drowsiness* is observed. The patient shrinks from all disturbance and shrieks out when roused sufficiently to move or perform voluntary acts. From this drowsiness, the step to coma and death is seldom many hours distant.—*British Med. Journal.*

**Experiments with Antiseptic Dressings.**—Dr. Bradford recently showed at a Boston Medical Society some specimens of meat done up in different dressings, and said that it was admitted that the main principle in surgical dressings consisted in the prevention of putrefaction of the discharges and dead organic matter about the wound. There had been a number of experiments as to the relative value of different antiseptics, but few, if any, as to the protective power of different dressings.

A piece of meat dissected from a freshly killed kitten and put into a bottle which had been rinsed out with carbolic acid (1 to 20) putrefied in three days. This was the case in a bottle corked and in one left open. A piece of fresh meat placed uncovered on a table at the side of the bottles dried up, and eleven days later gave no odor. A piece of flesh wrapped in water dressing became foul in three days. Flesh dissected out under spray and placed in a Lister dressing (antiseptic gauze), at the end of three weeks was found perfectly fresh not being at all dried. Flesh wrapped up in lint wet with terebene, at the end of three weeks was fresh and not dried. The same wrapped in lint wet with compound tincture of benzoin remained sweet, but dried. Labarraque's solution (1 to 10) poured on a cloth and wrapped about the meat delayed putrefaction but did not entirely prevent it. At the end of three weeks the meat was foul, but not so putrid as that in the water dressing. The same can be said of a solution of sulphite of soda 1 to 10. A mould was found on this dressing which, on examination, was found to be penicillum glaucum. Meat wrapped up in dry cotton cloth, Denison's absorbent cotton, oakum, salicylicized tow, or lint soaked in benzoic acid became desiccated. Meat not dipped in carbolic acid and covered with goldbeater's skin to prevent desiccation was not protected, when wrapped in cotton, from becoming at the end of three weeks slightly putrid. A piece of meat, however, placed in a glass tube, the end of which was stopped by cotton, without any treatment by carbolic acid, dried and remained free from putridity. The cotton in this case did not act by absorbing the fluid and desiccating, as it was so placed as not to touch the meat. Meat completely covered by fuller's earth and charcoal became dry, and remained free from odor. The piece of flesh which at the end of three weeks was the freshest, being as soft as when first dissected off, and entirely free from odor, was that wrapped in the antiseptic gauze. The next in excellence of preservation was that in lint wet with terebene. It has been stated that Lister's success is perhaps not due to any antiseptic (in the strict sense of the word) action of the dressing, but to the cleanliness his method enforces. As far as experiments on dead flesh can bear testimony, it seems evident that there is a strictly antiseptic action in Lister's gauze dressing.—*Boston Med. and Surg. Journal.*

**An Ulcer Nussbaumed.**—After a liberal trial of the grafting process, and a patient and concientious use of Esmarch's elastic bandage, the two being the latest novelties in the way of treating chronic ulcers, be they indolent or irritable, or both, I come back with more confidence to the operation suggested a few years ago by the distinguished surgeon Nussbaum. By it I have succeeded in curing these troublesome affections more surely, speedily and permanently than with any other plan.

The case before you is an indolent, irritable ulcer, involving the skin over the internal malleolus. It has been treated in a variety of ways; occasionally it seems upon the point of yielding; it grows less and begins to heal, but upon the slightest provocation it re-asserts itself. Upon exposure to cold, or after fatiguing exercise, the healed portion yields, the irritability returns, and the ulcer is soon of its original size—more indolent and painful. I now make an incision around it one-half an inch from its margin. This incision must go through the skin, it must reach the cellular tissue above the muscles. By it you divide the vessels, the numerous adventitious vessels developed in the peri-ulcerated skin that feeds the morbid process. In this way you cut off its direct and too liberal supply of blood. Into this canal you stuff lint and leave it there for twenty-four or thirty-six hours, long enough to prevent the severed tissue from rejoining by first intention. This is especially important with the arteries; they must not be allowed to unite.

In a short time the ulcer will begin to shrink; day by day the healing progresses, and in a week, or probably two, it will have disappeared.

This case was shown to the class three weeks after the operation. The ulcer had been healed, but a mistake had occurred in leaving the lint too long in the canal; it remained five days, and in that time suppuration had occurred in the floor. The repair in the canal required as much time as the ulcer.

The dressing after the operation is simple. Cold water for forty-eight hours, after that lint saturated with oil, simple cerate or vaseline.—*Cin. Lancet and Clinic.*

**Syphilis.**—In answer to "Can Syphilis be cured after it becomes constitutional, and how?" I have to say, It can; and in order to be brief will not enter into the pathology of the disease farther than to make the statement, it is a blood poison, and capable of being antidoted by a judicious application of the following treatment:

If the patient be robust, remove all his clothing, place him on a cane-seat chair, envelop him and the chair in a blanket, then place under the chair an alcoholic lamp beneath a flat basin containing a pint of hot water, and when the perspiration becomes profuse, light a second lamp, and place above the flame, on a tin sheet of copper, either the black oxide, red oxide, or sulphuret of mercury, according to the peculiar constitutional symptoms of the patient, and let it volatilize; by this time the patient will be in a profuse state of perspiration, and he will readily take 120 grains of mercury thus administered in one hour.

After the mercury becomes volatilized, which will take from seven to ten minutes, remove the last lamp and reduce the temperature beneath the blanket to 120°, letting the patient remain in the vapor for thirty or forty minutes. When the bath is over, sponge the body well and wipe dry, and when the body has regained its normal temperature, let him draw on his clothing, but not before, and so continue every four or five days until the syphilis has ceased, has entirely disappeared. If the patient be weak the bath must be more moderate at first, and his strength supported with iron, and any of the bitter tonics the physician may deem proper, in his peculiar condition. In conjunction I often use :

℞ Hydrag. chloridi.......... grs. i. to ij.
Pulv. opii................. grs. viii.
Pulv. guaici............... grs. xxxv.
M. ft. 10 to 20 pills. Sig ter die.

No ptyalization ever occurs in this course of treatment. I have had positive proof of the poison being entirely removed by this course several times, by the syphilitic taint in the children before treatment and its absence after treatment. It is necessary to add that both parents must be treated, where children have shown inherited syphilis. —*Dr. Allen, in Herald of Health.*

**Antiseptic Gauze.**—Lister's antiseptic gauze, which is prepared by impregnating a cotton-fabric of loose texture with a mixture of five parts rosin, seven parts paraffin, and one part of carbolic acid, has the disadvantage of being very stiff and unyielding. Dr. Paul Bruns, professor at Tubingen, proposes to overcome this difficulty by a change in the manner of impregnating the gauze, as well as by a different menstruum. He dissolves 400 grammes of powdered resin in 2 litres of alcohol, adds to the solution 40 grammes of castor oil, and finally 100 grammes of carbolic acid. The whole bulk measures now 2½ litres. This quantity is sufficient for impregnating 2 pounds (about 25 metres) of the gauze (previously deprived of grease). The gauze having been dipped into the liquid and well stirred about, it is removed, and suspended *horizontally* when it will dry in about half an hour. Thus prepared it is quite soft and pliable, and contains a 10-per-cent. solution of carbolic acid. After having been used, it may be cleansed by boiling in very dilute soda-lye, and then be impregnated again.

Improved benzoated or salicylated gauze or wadding has also been prepared by Prof. Bruns. Both of these heretofore suffered from the drawback that on handling they gave off a fine dust of benzoic or salicylic acid, which caused much annoyance to the operator or attendant. Prof. Bruns prepares it by adding 3 to 4 parts of castor oil to the solution—for every 10 parts of benzoic acid. 100 grammes of benzoic acid and 40 grammes of castor oil (or 20 grammes each of castor oil and resin), are dissolved in 2.56 litres (2.560 cc.) of alcohol, the gauze soaked in the liquid, and then dried. This gauze contains a 10-per-cent. solution of benzoic acid. The salicylicated gauze is prepared in the same manner.—*New Rem.*

**Apiol,** the active principle of parsley, discovered by Taret and Homelle in 1855, is an oleaginous amber-colored liquid, soluble in water in any proportion, and of an acrid taste. It may be given in capsules. According to Marotte, of La Pitie, it brings on the menses, regulates menstruation, and calms the pains by which it is often accompanied. It has no action on the pregnant womb.—*Maryland Medical Journal.*

**How to Avoid Leaving Scars.**—At a conversational meeting of the Philadelphia County Medical Society, Nov. 13th, 1878, it was addressed by Dr. John H. Packard on "Some Surgical Wrinkles." The first point he discussed was a method of making superficial incisions by which scaring can be avoided. In operations upon exposed parts, such as the face and hand, it is very desirable that they shall be so done as to leave as little scar as possible. The procedure recommended was first suggested by witnessing the effects of an accident, a lady having fallen while carrying a china dish, a piece of which made a long gaping incised wound in her hand, the sharp knife-like edge having cut through the skin very obliquely. The wound healed readily, almost without a scar. A few weeks after the traces of the injury could scarcely be discovered.

Thinking that this effect was in a great measure due to the direction of the incision through the skin, the speaker tried the experiment in cutting down upon a tumor of the thigh, holding the knife so as to divide the skin obliquely. The wound united perfectly, and after it had healed he actually could not find the line of incision. Since that time he had tested the idea in numerous other cases with highly satisfactory results. In small superficial operations, such as the removal of small tumors from the face, it has a cosmetic advantage that at once recommends it.—*Cin. Lancet and Clinic.*

**Injurious Effect of Use of Tobacco on the Eye.**—Dr. Chisolm, in referring to cases from this cause, speaks of the treatment as follows: He has found but one remedy against tobacco poisoning, viz: total abstinence from the use of the weed. Nothing short of this will restore sight to the dim eye. He had seen cases where strychnia and electricity had been used in vain until the cigar was laid aside, when improvement commenced to show itself at once, and continued rapidly to complete restoration. He believes that the discarding of tobacco alone would cure many cases. He usually aids the restoration by means of strychnia. His minimum dose to an adult is one-twentieth of a grain sulphate of strychnia to be taken after each meal. This dose he slowly increases to one-fifth grain, which most patients can comfortably bear in time, so rapidly does the system accommodate itself to the drug. After eating is the proper time to administer the dose. He had experimented extensively with strychnia in the hypodermic injection, the fluid dose by the mouth, and the sugar-coated granules. He uses exclusively the latter, as equally effective and most agreeable.—*Maryland Med. Jour.*

**Chronic Bright's Disease.**—Dr. Richardson, in the *Philadelphia Med. Times*, says this disease is so intractable in nature as to be a matter of universal experience, and that even a faint hope of greater success in its cure is worthy of consideration. One chance of successful treatment seems to lie in cutting off the supply of fat (for example, by an exclusive skim-milk diet), at the outset of the attack; and for this an early diagnosis of incipient fatty degeneration is necessary. Frequent observation of the remarkable power which osmic acid has of blackening, and thus detecting extremely minute globules of fat, suggested the idea that this agent might be advantageously employed in recognizing fatty metamorphosis in its commencement, and on trial it has been found that osmic acid does enable us to discover in epithelial cells particles of oil so small as ordinarily to elude detection. The acid solution in water should be used, of the usual one per cent. strength, and this preparation, when secluded from the light, keeps perfectly well for months, and even years. Its value in recognizing fatty degeneration elsewhere, as, for example, in the voluntary muscles, the heart, etc., is obviously great.

**Intestinal Gas---Flatulent Dyspepsia.**—Dr. Eads (in *Eclectic Med. Journal*) in a case of the above character prescribed:

Tinct. colocynth........... gtt. x.

Water...................... ℥ iv.

He gave a teaspoonful every three or four hours until relieved. Next day he found his patient perfectly free from pain, no wind in stomach or bowels—the first permanent relief obtained for two months. The treatment was continued for a few days only; the patient thinking herself well, discontinued it. In a short time the flatulency returned, but not as severe as before. Ordered the above prescription to be taken whenever there is any gas in the bowels or stomach. Pregnancy was now in the eighth month. Hoping there would be no further trouble after confinement, that time was looked for with great interest.

Jan. 10th, 1878, delivered patient of a fine 8-℔ male child; recovery after confinement good, even better than was expected; considerable trouble the first three weeks with intestinal gas, but relieved every time with colocynth. When the babe was about three weeks old it began to suffer very much like the mother with an accumulation of intestinal gas.

Colocynth .................. gtt. ij.
Water....................... ℥ ij.

A teaspoonful three times a day gave prompt relief. Now, nine months since, mother and child both well. I believe colocynth to be a specific in the above condition.

**Electricity for Sleeplessness.**—That galvanization of the head has an hypnotic effect, has long been known; hitherto, however, it has not been used to counteract sleeplessness. Vigoureux asserts (*Allg. Wiener Med. Ztg.*) that he has daily obtained the finest results in this direction, and has failed only in exceptional cases, as, for instance, when sleep has been disturbed or prevented by severe dyspnœa. His method is to place the broad flat electrodes (carbon covered with chamois-leather) on both temples, and allow the current of from three to at the most five Trouve's elements pass for a half or a whole minute. When the application is made in the morning, the patient experiences a more or less pronounced inclination to sleep. Occasionally the effect of the galvanization is prolonged after the first night, for a night or two. —*N. Y. Med. Journal.*

**Hooping Cough and Ulceration of the Phrænum.**—A recent report before the Academy of Medicine in Paris, says that the ulceration is an effect rather than a cause of the disease, and is due to the protrusion of the tongue and the scraping of its under surface against the teeth, in the act of coughing.—*Med. Record.*

**Vinegar Fumes in Bronchitis, Etc.**—Dr. Richardson, in the Philadelphia *Medical Times*, calling attention to the histological revival of motion in cilia and ciliated cells when exposed to the action of dilute acetic acid, or to the fumes of vinegar, suggests that the latter may be a potent stimulus to cilia in their normal position on the mucous membrane. As this wave of ciliary movement has so much to do in bringing out the mucus, (which, in bronchitis and similar diseases, is poured forth to excess), and thus freeing the clogged-up air-passages, we have both an explanation of the old woman's cure for a cough in hot vinegar, and a strong incentive to use it—in spray from an atomizer, or otherwise—more persistently and systematically in bronchitis and allied disorders. It would also guide us to employ it in opium-poisoning, in paralysis, and in all prostrating diseases, when the lungs begin to fill up, probably in part as a consequence of enfeebled ciliary movement.—*St. Louis Courier of Medicine.*

**Treatment of Tetanus.**—Dr. Aribaud reports a number of cases of traumatic tetanus in which he employed a mixed treatment of chloral and opium. The fatal terminations being apparently delayed by giving the remedies in increased doses, the doctor finally arrived at a somewhat definite line of treatment by means of which he has saved three out of his last four cases.

From his experience, he states that a daily dose of six to seven grammes of chloral must be given continuously, until the patient is almost well, as in less quantities the remedy seems to have no serious effect upon the course of the disease.

The opium was administered under the form of laudanum, forty drops of which were given daily by means of rectal injections. He preferred the use of enemas, as he could thus confide the administration of the opium to the care of any assistant. Attention should of course be paid to the physiological effects of both remedies.—*Lyon Medicale.*

**Treatment of Gingivitis, or Sore Gum, in Pregnant Women.**—By PINARD.—Hydrate of chloral and tincture of cochlearia officinalis (common scurvy grass), equal parts. This solution was applied every day, or every two days, to the free and diseased edge of the gums by the aid of a mop. This dressing was slightly painful, and the cauterization very light. The white and very superficial eschar disappeared generally twenty-four or thirty-six hours after the application. In thirty women attacked with gingivitis, who were subjected to this treatment, twenty-five were cured in less than fifteen days. In two of them the cure was slower, complications having supervened which necessitated the use of ointments and mercurial ointment. In the five others, the treatment could not be continued.—*L'Abeille Medicale.*

**Salicylic Acid as an Antiseptic and Antipyretic.**—In 1876, Mr. Prideaux treated with salicylic acid, or its salts, eighty-eight cases of confluent small-pox, in the Derby Hospital, without losing a case. Since then, seventy-eight cases of scarlet fever, with only one death, and that a case seen at the height of the disease. Many cases of measles, and some few attacks of typhoid fever, yielded equally satisfactorily to the same treatment.—*London Med. Rec.*

**Treatment of Cold Feet when causing Sleeplessness.**—Putting them to the fire gives temporary warmth, and so does the hot bottle in bed, so long as it remains itself hot; but as it cools the feet again become cold, and sleep could not be wooed successfully. What should be done is to dip the feet momentarily into cold water, and then have them well rubbed with hair gloves or a rough towel until they glow. This seems a very unattractive plan to many minds; but it is just the story of the snowballer's hands. At first the contact of the snow makes the fingers very cold; but perseverance is rewarded by a glow which may become almost a burning heat; the primary contraction of the vessels is followed by a secondary dilatation. This is what we will accomplish by the immersion, for a brief period only, of the feet in cold water, followed by friction. By such means the cold feet become warm, and after this a hot bottle to the feet will keep them warm effectually.—*Dr. Fothergill, in Med. News.*

**Evacuation of Pus from the Pleura by Inversion of the Body.**—Dr. Raynaud has tried with success the following method: A girl, fifteen years of age, convalescing from typhoid fever, contracted a purulent pleurisy, and after a time there was pulmonary perforation followed by a considerable vomica. The expectoration was insufficient to empty the liquid contained in the pleura, and in consequence the general condition became constantly worse. Dr. Raynaud then placed the child with her head below the border of the bed, and this manœuvre was followed by an abundant expectoration. This process, repeated several times, emptied the pleura of its purulent contents, and the child rapidly recovered its strength and was soon quite well.—*Giorn. Ven. di Sc. Med.*

**Polypi of the Ear.**—These troublesome growths, which, as I have said, are almost invariably accompanied by an otorrhœa, generally very profuse and offensive in character, are, as a rule, easily gotten rid of. They may be readily removed, most conveniently by Blake's modification of Wilde's snare, or by the canula forceps, (an instrument which does not seem to be as generally known among aural surgeons of the present day as it should be) and though they are prone to recur, if the treatment cease with their removal, this tendency may be overcome by the subsequent insufflation of powdered alum, or by repeated instillation of a solution of sulphate of zinc. The application of chromic acid to the remains of the pedicle is also a convenient and efficacious means of accomplishing the same end.—*Mar. Med. Journal.*

**Turpentine-Vapor Baths.**—Turpentine-vapor baths in rheumatism are highly recommended for the chronic form. Place the patient in a wooden chest, allowing the head to remain free. Conduct into this wooden chest steam loaded with oil of turpentine spray. The temperature of the bath should be 104° F. to 113° F. Let the patient remain therein about fifteen minutes. Repeat them according to the nature of the case.—*Med. Record.*

**Subnitrate of Bismuth.**—Commercial subnitrate of bismuth almost always contains lead. The quantity varies from a mere trace to a dangerous proportion.—*Phila. Drug. and Chem.*

**Treatment of Articular Rheumatism by Benzoic Acid.**—The author employed this new therapeutic agent twenty times in acute, and ten times in chronic articular rheumatism. In the latter cases the results obtained were not very satisfactory, which is not surprising as chronic rhumatism offers small encouragement for treatment. Benzoic acid has the advantage of salicylic acid in that it does not produce bad impressions in ordinary doses—at the same time having a sufficiently rapid effect in checking the disease. It is to be observed, however, that the temperature decreases less rapidly, no matter in what disease it is used.

The dose in simple cases is from eight to twelve gms. (grs. 120 to 130) or even more in the adult. The author fails to communicate in what vehicle it should be exhibited. The curative action of benzoic and salicylic acid is probably due to the property they possess of arresting pathological fermentation of the tissues or in the circulatory stream. —*Mar. Med. Journal.*

**Syphilitic Laryngitis.**—Duret suggests the following methods of treatment in the course of a review of M. Isambert's works on "Syphilitic Laryngitis." The general treatment should consist in protiodide of mercury in pills (¼ to ⅓ grain three times a day) or bichloride in solution. Should the affection have passed the secondary stage, iodide of potassium may be employed; or in stubborn cases, the "mixed treatment." Tonics—iron, quinine, etc.—are usually called for. The patient should avoid the use of tobacco and alcoholic liquors. Complete rest of the organ is essential.—*Ex.*

**Charcoal for Burns.**—A venerable patient, a retired foundryman, tells us that during his apprentice-hood to a ship-builder of Philadelphia, he became acquainted with a never-failing remedy for burns and scalds, and that in his subsequent foundry-life he saw innumerable such injuries relieved of pain, and healed as if by magic, by powdered charcoal. The softer it is the better, and that from pine wood is the best. It is to be thickly sprinkled over the burnt or scalded surface as soon as possible, and renewed as it becomes moist or drops off.—*Louisville Med. News.*

**Horrible!**—Dr. Leidy, of Philadelphia, has announced the discovery that cucumbers are liable to be infested with tape-worm. At a meeting of the Academy of Sciences, Philadelphia, he exhibited a specimen of tape-worm taken from the inside of a large cucumber. It is said to have had all the characteristics of a true tape-worm, but belonged to an unknown species, the peculiarity being that the ovaries containing the round yellow eggs are confined to the anterior extremity of the segment.—*Ex.*

**Atropia and Daturia are not Identical.**—According to V. Planta's investigations, atropia and daturia were hitherto regarded as identical. Poehl's new researches (*Petersb. Med. Wochensch.*, 1877, No. 20) have, however, shown that they are chemically different. Atropia is optically inert; daturia turns the plane of polarization $14.12^{\circ}$ to the left. Atropia salts are precipitated by platinic chloride, those of daturia are not; picric acid precipitates daturia, but not atropia.—*Pharm. Centralh.*

**Cholera Infantum.**—During the summer of 1873 I was called to prescribe for a child two years old, supposed by the physician in attendance to be dying, the disease being diagnosed as cholera infantum. My prescription was one ripe strawberry every hour till better. The child speedily recovered. Three months after, I was asked to prescribe for another child aged eleven months. The disease this time was really cholera infantum. One-half strawberry every hour proved a successful treatment. This child had also been given up to die.—*Boston Journal of Chemistry.*

**Carbolic Acid Spray in Diphtheria.**—Dr. Booker, in the *American Practitioner*, reports a number of cases treated in this way; he says:

"The characteristic membrane was to be seen in the fauces in each case. The treatment ordered in these cases was quinia and tincture of chloride of iron, to be given every two hours in doses of three grains of the former to five drops of the latter; and carbolic acid, one part to twenty of water, to be applied hourly to the fauces with the spray producer. These cases recovered rapidly."

**Blepharitis.**—Dr. Roy ordered, in a stubborn case, a cotton compress, covered with caoutchouc. By mistake, the mother of the child applied the patches of rubber directly to the diseased lids. They were kept on over-night for two weeks before Dr. R. was again consulted, when, to his astonishment, he found that the blepharitis was entirely cured. He has since used this simple application in a number of cases, and always with success. The rubber patch is simply bound over the eye through the night, and the lids washed with warm water and soap in the morning.—*Bull. Gen. de Therap.*—*Med. Times.*

**Benzoated Cotton-Wool.**—Prof. Volkmann, of Halle, who deserves the highest credit for the remarkable success he has achieved in antiseptic surgery, uses, among other means of protecting wounds, benzoated cotton-wool (three per cent. and ten per cent. of benzoic acid. It is generally used as an external dressing, but in some cases, especially in dressing small wounds, it is applied directly to the surface, and covered with an elastic flannel bandage.—*N. Y. Med. Jour.*

**Diagnosis of Aneurism in Aorta.**—W. S. Oliver, M.D., Surgeon-Major, says that he has been materially assisted in diagnosing *Aneurism* of the aorta, when it occurs, as is most generally the case, either in the ascending or first part of the transverse portion of the arch, by the following process: Place the patient in the erect position with closed mouth, and extreme elevation of the chin; then grasp cricoid cartilage between the thumb and finger, and press it gently upwards, when, if aneurism exist, the pulsations of the aorta will be distinctly felt.—*London Lancet.*

**Insomnia.**—In cases of insomnia from anxiety, melancholia, or a low state of nutrition, camphor will act favorably when chloral, morphia, potassium bromide, etc., fail. One and a half to two or three grains may be subcutaneously injected after dissolving in almond oil.—*N. Y. Med. Record.*

**Fibroid Tumors of the Uterus Treated by Sclerotic Acid.** Dr. John Williams reports two cases of uterine fibroid tumor treated by sclerotic acid. This acid dissolves readily in water, and in so far differs from ergotine. One of the cases was that of a woman aged thirty-four, who had suffered from severe flooding for some time. A fibroid tumor was detected, and half-grain doses of the acid were injected under the skin of the abdomen twice a week. The flooding was much reduced, but returned during a temporary discontinuance of the treatment. When the injections were again commenced, the flooding was checked as before. The tumor was reduced in size. Like results were attained in a similar case, including a decrease in the size of the tumor. The injection caused a little pain at the time, but that was all. It was followed in about half an hour by uterine contractions. *British Med. Jour.*

**Codeia for Cancer of Stomach.**—Dr. Austin Flint says: With reference to treatment, it is merely palliative. Pain in this instance is a symptom to be palliated; so also is the vomiting. I should say, prescribing for the patient as now presented, that some form of opium is indicated. I prefer to use codeia, for the reason that it is much less liable to produce disagreeable after-effects than are some of the other preparations of opium. I should recommend, therefore, that this man take ¼ of a grain of codeia twice a day.

**Diphtheria.**—After all that has been written, and all the thousand and one formulæ that have been proposed for diphtheria, nothing has been found to excel the chlorate of potash.

It should be given *in saturated* solution in one to two teaspoonsful doses every one to two hours.

Sulphate quinine, muriated tinc iron, or other appropriate tonics may be combfned according to the circumstances of the case.

**Treatment of Ulcers.**—Dr. Mandelbaum asserts that ulcers of the leg which have proved rebellious to all other methods of treatment, can be invariably cured by scraping, followed by the application of iodoform which must be continued for several days, and, after fresh granulations have appeared, by firm pressure with equal parts of mercurial and soap plaster.—*Berlin Klin Wochen.*

**Quinine a Dangerous Oxytocic.**—Mr. Parak claims that in a number of experiments he has found this remedy constantly in the urine of the fœtus, even when it is administered in seven and a half grains, an hour before delivery, it having passed through the placenta. Icterus he has observed as a common effect of the quinia on the fœtus.

**Treatment of Gonorrhœa.**—Lecchini recommends for acute gonorrhœa, two injections daily of a solution of chloral hydrate (1 : 100). He claims that it relieves rapidly the most troublesome symptoms, and even effects a cure after only a few injections.—*Deutsche Med. Wochen.*

**Tincture of Veratrum.**—Tincture of veratrum will be found a valuable remedy in curing whitlow and felons.—*Ibid.*

